# A primer on burn resuscitation

**DOI:** 10.4103/0974-2700.76845

**Published:** 2011

**Authors:** Ferdinand K Bacomo, Kevin K Chung

**Affiliations:** U.S. Army Institute of Surgical Research, 3400 Rawley E. Chambers Avenue, Fort Sam Houston, TX 78234, USA

**Keywords:** Burn, creep, fluid, formulas, resuscitation, rule of 10, shock, ten

## Abstract

Since the early 1900s, the scope of burn resuscitation has evolved dramatically. Due to various advances in pre-hospital care and training, under-resuscitation of patients with severe burns is now relatively uncommon. Over-resuscitation, otherwise known as “fluid creep”, has emerged as one of the most important problems during the initial phases of burn care over the past decade. To avoid the complications of over-resuscitation, careful hourly titration of fluid rates based on compilation of various clinical end points by a bedside provider is vital. The aim of this review is to provide a practical approach to the resuscitation of severely burned patients.

## INTRODUCTION

Since the early 1900s, advances in burn resuscitation have significantly reduced morbidity and mortality.[1] Numerous formulas have been vital in guiding clinicians through the initial resuscitation of the severely burned and due to various advances in pre-hospital care and training, under-resuscitation of patients with severe burns is now relatively uncommon. “Fluid creep”, a term originally coined by Dr. Basil Pruitt to describe the phenomenon of over-resuscitation, has emerged as one of the most important problems during the initial phases of burn care over the past decade.[2–5] It appears that one reason may be due to many clinicians finding current formulas to be too cumbersome to follow.[3]

In this review, a concise practical guide for clinicians involved in leading a resuscitation of the thermally burned will be provided. In addition, we review the current controversies and the application of potential adjunctive therapies when faced with a difficult resuscitation in an effort to avoid “fluid creep”.

## PATHOPHYSIOLOGY OF BURN SHOCK

Typically, patients are at risk for developing burn shock if they have greater than 20% of their total body surface area (TBSA) burned. Factors such as the depth and extent of the burn, pre-existing illness, and the presence of inhalational injury greatly influence the magnitude and duration of shock.[6] Burn shock physiology can be divided into two phases; the emergent phase and the flow phase.

The initial phase of burn shock, also known as the emergent or ebb phase, is at maximum 12 h into the postburn and usually lasts for up to 72 h. This phase of injury is characterized by a combination of increased capillary permeability and cellular changes. Capillary permeability is a direct result of histamine, prostaglandins, and other vasoactive substances released into circulation.[7] The capillary permeability also causes a decrease in interstitial oncotic pressure as proteins are lost into the interstitium. Cellular changes occurring in burn edema include a decrease in cell transmembrane potential, leading to increased cellular swelling from the influx of sodium.[8] Overall, these changes lead to massive fluid shifts from the interstitium to the intracellular space of both burned and nonburned tissues.[9] Additionally, there are direct cardiac effects in the setting of burn shock. A decrease in contractility leads to a depressed cardiac output, which is believed to be the hallmark of the early postinjury phase. This is felt to be a result of circulating mediators (i.e., TNF-α) and impaired intracellular calcium ions.[10]

## GOAL OF BURN RESUSCITATION

The primary goal of fluid resuscitation after severe burn is to maintain adequate tissue perfusion to the end-organs with intravenous crystalloid in an effort to avoid ischemic injury at the lowest physiologic cost.[11] Extreme difficulty of this task is related to the pathophysiologic changes described which occur during the emergent or ebb phase immediately postburn. The profound capillary leak combined with the potential for cardiac compromise leading to hemodynamic collapse makes adequate resuscitation of these patients extremely difficult.

## CONSEQUENCES OF UNDER-RESUSCITATION

Delayed or inadequate replacement of intravascular volume results in suboptimal tissues perfusion. Suboptimal tissue perfusion results in end-organ damage that may become irreversible and ultimately contributes to death. Fortunately, under resuscitation is relatively uncommon since the adoption of weight and injury-based resuscitation formulas. On occasion, however, the patient can develop a concurrent distributive shock which necessitates additional volume to maintain adequate perfusion.

## CONSEQUENCES OF OVER-RESUSCITATION

**Figure 1:**
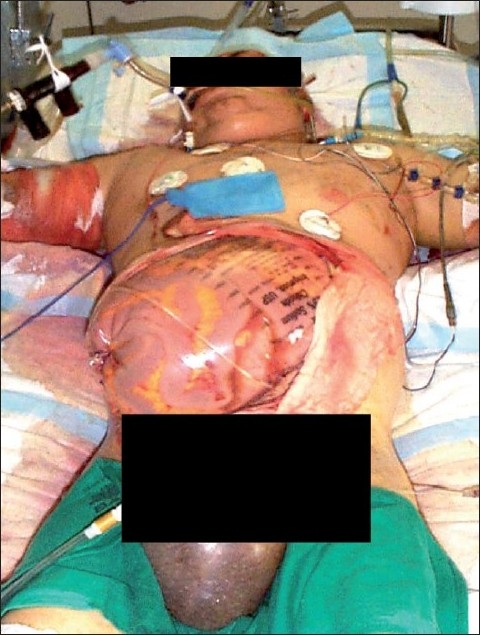
Burn patient after a decompression laparotomy for abdominal compartment syndrome

Over-resuscitation often results in “resuscitation morbidity”. Resuscitation morbidity is a term used to describe complications of fluid overload which includes orbital compartment syndrome, extremity compartment syndrome, pulmonary edema, and abdominal compartment syndrome.[12,13] Of these, the most dramatic and clinically challenging is the development of abdominal compartment syndrome [Figure 1]. A resuscitation volume greater than 237 cc/kg over the course of 12 h (or 16 L during a 12-h period in a 70-kg man) appears to be the threshold for the development of ACS.[14] In our own experience, resuscitation-related abdominal compartment syndrome is associated with a mortality of 97% when burn size is greater than 60% TBSA.[15]

## RESUSCITATION FORMULAS

Fluid resuscitation with crystalloid is the cornerstone of initial burn management. Various formulas have been developed over the years that estimate the fluid requirements in the first 24–48 h [Table 1]. Perhaps the most commonly used formula is the Parkland formula which estimates 4 mL/kg/% TBSA of total volume in the first 24 h after burn injury. From the total fluids calculated, it is estimated that half will be administered over the first 8 h, and the rest given over the next 16 h. A number of recent authors have recently reported that this formula significantly underestimates actual fluids received.[2,4,16,17] Other authors have noted that that the Modified Brooke formula, which estimates a total volume of 2 mL/kg/%TBSA over a 24 h period, also underestimates the actual resuscitation volumes received.[18] In fact when the two formulas were compared head-to-head in a retrospective case controlled study, the higher the initial rate, the higher the final resuscitation volume at 24 h.[4] In this report, those who had a resuscitation based on the Modified Brooke formula group ended up requiring approximately 3.8 mL/kg/%TBSA; while those were started on fluid rates based the Parkland formula ended up requiring approximately 5.9 mL/kg/%TBSA (*P* < 0.0001). A greater percentage of patients in the Parkland group exceeded the Ivy Index, defined as 24 h volumes exceeding 250 mL/kg (57% vs. 29%, *P* = 0.043).[19] This was found to be an independent predictor of death based on multivariate logistic regression. No difference was detected between the two groups in terms of other clinical outcomes such as the incidence of lung injury, kidney injury, and ACS, as well as ventilator days, ICU days or death. Many authors agree that the most important variable during the resuscitation of a severe burn patient is the careful bedside titration of the hourly fluid based on the compilation of various clinical end points. Thus, it is reasonable to believe that the starting point is almost irrelevant due to the complex nature of the body’s response to burn injury.

**Table 1:** Burn resuscitation formulas

1942	Harkins formula	Any patient with at least a 10% burn: administer 1,000cc plasma for each 10% total surface area burn over first 24hrs.
1947	Body weight burn budget	First 24 hrs: 1-4 L LR + 1200ml 0.5NS + 7.5% body weight colloid + 1.5-5L D5W.
		For second 24hrs: same formulation except change colloid to 2.5% body weight
1952	Evan’s formula	First 24hrs: NS at 1ml/kg/%burn + colloids at 1ml/kg/%burn + plus 2000ml glucose in water.
		Second 24hrs: one-half the first 24hrs crystalloid and colloid req + the same amount of glucose in water as in the first 24h.
1953	Brooke formula	First 24hrs: LR at 1.5 ml/kg/% TBSA burn + colloid at 0.5 ml/ kg/% TBSA burn.
		Second 24 hrs: Switch to D5W 2000 ml.
1974	Parkland formula	First 24 hrs: LR at 4ml/kg/%TBSA; give half in first 8 hrs and the remaining over next 16 hrs.
		Second 24hrs: colloid at 20-60% of calculated plasma volume to maintain adequate urinary output.
1979	Modified brooke	First 24 hrs: LR at 2 ml/kg/% TBSA burn, one half in the first 8 hours and half in the remaining 16 hours.
		Second 24 hrs: colloid at 0.3 to 0.5 ml/kg/% TBSA burn + D5W to maintain urine output.
1984	Monafo formula	First 24hrs: Saline with 250 mEqNa + 150 mEqlactate + 100 mEqCl. Rate adjusted per urine output.
		Second 24 hours: one third of isotonic salt administered orally.

As a result, the approach in our burn center has been to derive the initial fluid rate using a simplified formula called “the Rule of 10”.[20] The Rule of 10 consists of three steps [Table 2]. The first step is to estimate the burn size (% TBSA), to the nearest 10. Second, that number is multiplied by 10 to derive the initial fluid rate in mL/h. For every 10 kg above 80 kg, add 100 cc/h to this rate. Once initiated, the ultimate goal of burn resuscitation is to provide the least amount of fluid necessary to avoid end-organ failure while avoiding the pitfalls of “fluid creep.”[21] This simple formula allows providers to rapidly determine the initial rate in adults (>40 kg) and allows the emphasis to be placed, appropriately, on the process of resuscitation. As recently validated in an *in silico* analysis of 100,000 simulated “patients”, the Rule of 10 derives a reasonable starting point for a wide range of burn sizes and adult patient weights (40–140 kg).[14]

**Table 2:** The rule of 10

1	Estimate burn size to the nearest 10
2	%TBSA × 10 = Initial fluid rate in mL/h (for adult patients weighing 40–80 kg)
3	For every 10 kg above 80 kg, increase the rate by 100 mL/h

## PEDIATRIC RESUSCITATION

It is important to emphasize that this method of deriving the initial fluid rate should not be applied to pediatric burn patients. In a recent guideline, the American Burn Association recommended that pediatric patients with thermal burns are resuscitated with initial fluid rates derived by the Parkland formula.[22] In addition, due to rapid depletion of glycogen stores in fasting children, sufficient glucose substrate is required in the first 24 h of the resuscitation. This can be achieved by the addition of dextrose containing maintenance fluid to the resuscitation fluid or by initiation of early enteral feeds.

### Flow-sheet

**Figure 2:**
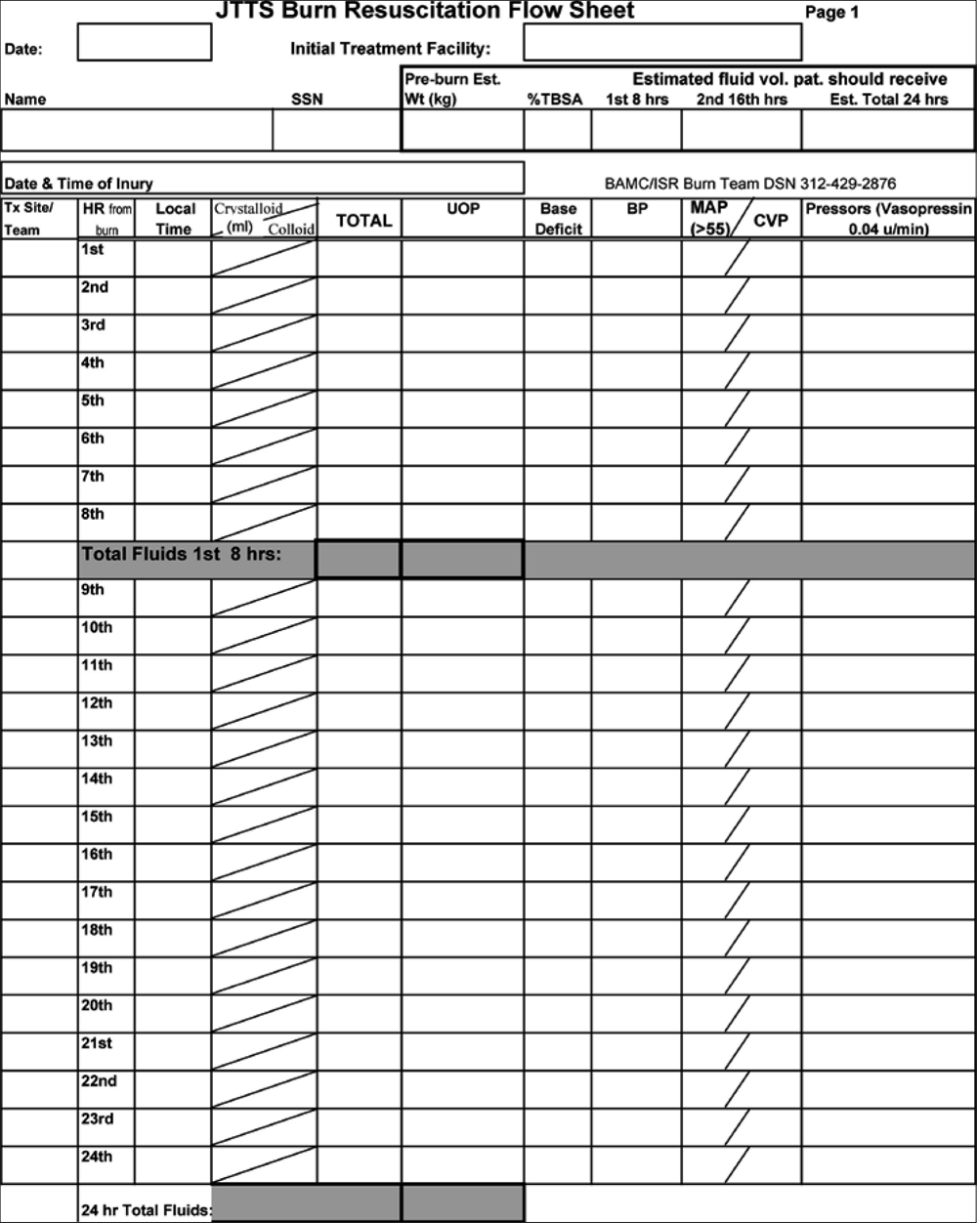
Sample flow-sheet used by the US military for standard documentation of burn resuscitation

Once the fluid resuscitation is initiated at a given rate, emphasis must be placed on the ongoing resuscitation by attentive bedside care providers with close monitoring of hourly urine outputs, base deficit, mean arterial pressure and if a central venous line is available, central venous pressure. Fluids rates should be titrated, increased or decreased, hourly based on a compilation of various clinical endpoints centered on a target urine output of 0.5–1 mL/kg/h or approximately 30–50 mL/h. In children, the target urine output should be closer to 1 mL/kg/h. In general, titration of fluid rates up or down at each point should not exceed 30% of the current rate in an effort to avoid wide fluctuations. Within the US military, providers are asked to carefully track fluid resuscitation on a burn-specific resuscitation flow-sheet during global evacuation back to our burn center in Texas [Figure 2]. Implementation and compliance with this burn flow-sheet along with resuscitation guidelines have made a substantial impact in reducing resuscitation morbidity and improving mortality in those being primarily resuscitated by nonburn providers.[12,23] In the very near future, automated computerized decision support systems may be available to guide nonburn clinicians through a difficult resuscitation on the basis of the capture of continuous physiologic feedback along with automatic recording of resuscitation data.[24]

### Adjunctive therapy

Despite timely and adequate fluid resuscitation, many patients with severe burns will develop refractory burn shock leading to a “runaway” resuscitation. As such the following adjunctive therapies may be required.

## ALBUMIN RESCUE

Despite some reservation with the use of albumin in the early phases of burn resuscitation, recent work by Cochran *et al*. demonstrates a decreased likelihood of death.[25] In a recent case controlled study, Lawrence *et al*. demonstrated that the addition of colloid to crystalloid resuscitation rapidly reduced hourly fluid requirements and ameliorated fluid creep.[26] We routinely administer albumin as early as 12 h postburn in patients in whom we project a “runaway” resuscitation at 12 h [Table 3]. In the previous study mentioned, 40% of all patients received 5% albumin after 12 h.[4] In select patients, after carefully weighing risk and benefit, fresh frozen plasma may play a role in decreasing resuscitation morbidity. In a prospective randomized trial, O’Mara *et al*. demonstrated decreased overall fluid volumes and lower intra-abdominal pressures when comparing a crystalloid resuscitation versus one supplemented with fresh frozen plasma.[27]

**Table 3:** Guidelines for the difficult resuscitation

At 12–18 h post-burn, calculate the PROJECTED 24-h resuscitation if fluid rates are kept constant. If the projected 24-h resuscitation requirement exceeds 6 mL/ kg/%TBSA or 250 mL/kg then the following steps are recommended Initiate5%albuminatarateof25-100ml/hr.(20-30%=25ml/hr,31-44%=50ml/hr,45-60%=75ml/hr,<61%=100ml/hr) Check bladder pressures every 4 h. If urine output (UOP) < 30 cc/h, consider monitoring central venous pressures (CVP) from a subclavian or IJ along with central venous (ScvO2) saturations. (Goal CVP 8–10, ScvO2 60–65%) If CVP not at goal then increase fluid rate. If CVP at goal then consider vasopressin 0.04 units/min to augment MAP (and thus UOP) or Dobutamine 5 mcg/kg/min (titrate until SvO_2_ or ScvO_2_ at goal). Max dose of Dobutamine is 20 mcg/kg/min. If both CVP and ScvO_2_ at GOAL then stop increasing fluids (EVEN if UOP < 30 cc/h). The patient should be considered hemodynamically optimized and the oliguria is likely a result of established renal insult. Some degree of renal failure should be tolerated and expected Continued increases in fluid administration despite optimal hemodynamic parameters will only result in “resuscitation morbidity”, that is oftentimes more detrimental than renal failure. Every attempt should be made in minimize fluid administration while maintaining organ perfusion. If UOP > 50 cc/h, then decrease the fluid rate by 20%. After 24 h, LR infusion should be titrated down to maintenance levels and albumin continued until the 48 h mark.

### Vitamin C

The development of oxidative stress is a major element in burn pathophysiology. Burn injury induces the formation of reactive oxygen species, which distorts the balance between radical formation and elimination. As Pham *et al*. were able to exhibit in burned mice, burn-mediated changes in the liver increase perioxidation and decrease antioxidant capacity.[28] Foldi *et al*. were able to demonstrate in a study analyzing the oxidative stress response after severe burn injury that the type of fluid resuscitation has only a moderate effect on the pro-oxidant state and does not influence the changes of endogenous antioxidants in burned patients.[29]

Using high dose ascorbic acid, 66 mg/kg/h for 24 h, Tanaka *et al*. found that adjuvant ascorbic acid administered during the first 24 h after thermal injury significantly decreased fluid administered compared to control (3.0 vs. 5.5 mL/kg/%TBSA, respectively, *P* < 0.01).[30] High dose vitamin C therapy may be considered as an adjunct in those at risk for fluid overload.

### Plasma exchange

Originally described almost 30 years ago, the plasma exchange is thought to be effective in removing the circulating mediators responsible for the phenomenon of burn shock.[31] According to a study performed by Neff *et al*. when therapeutic plasma exchange was utilized as a salvage maneuver, significant physiologic improvement and cessation of increasing fluid rates occured. Also, both MAP and UOP greatly increase while lactate levels decrease.[32] Prospective data are lacking to widely apply this method for patients at risk for over-resuscitation.

### Hemofiltration

Another method of extracorporeal blood purification is hemofiltration. Clearance of molecules of middle molecular weight (up to 50 kDa) is possible *via* convection as delivered by hemofiltration.[33] Our group recently demonstrated an absolute reduction of 33% and 23% in 28-day mortality and hospital mortality in critically ill burned military casualties with acute renal failure (*n* = 29) aggressively treated with high volume continuous venovenous hemofiltration (CVVH) when compared to a closely matched historical cohort (*n* = 28).[34] In a subgroup of patients with shock (*n* = 21), a majority of them with septic shock, we observed a dramatic reduction in the vasopressor requirement at 24 and 48 h that did not exist in the historical cohort (*P < 0.05 both to baseline and between groups). Due to potential effects in reversing shock, we believe this mode of therapy may have a potential role as a salvage maneuver in patients with burn shock undergoing a “runaway” resuscitation.

## CONCLUSION

Optimal care for the seriously burned patient should start with prompt recognition and evaluation of the burn. Once that has been established, resuscitation should be initiated via a simple formula right away. The initial fluid rate is not as important as the actual resuscitation itself guided by an attentive bedside clinician. Fluids should be titrated based on a compilation of various end points with the main goal of maintaining urine output 0.5–1 cc/kg/h, or roughly 30–50 cc/h. Best results are obtained by tracking the resuscitation on a separate flow-sheet. The need to define better endpoints of resuscitation to avoid excessive volume administration represents a high priority for future investigations. Additionally, the role for various adjunctive and salvage therapies when anticipating a “runaway” resuscitation must be carefully considered weight risk and benefit. Many of these adjuncts will need to be defined more clearly by future clinical trials.
